# Supplementary figures and images for: Cytokine induced inflammatory bowel disease model using organ-on-a-chip technology
Source: PLoS One. 2023 Dec 13;18(12):e0289314. doi: 10.1371/journal.pone.0289314 (PMC10718466; doi:10.1371/journal.pone.0289314)

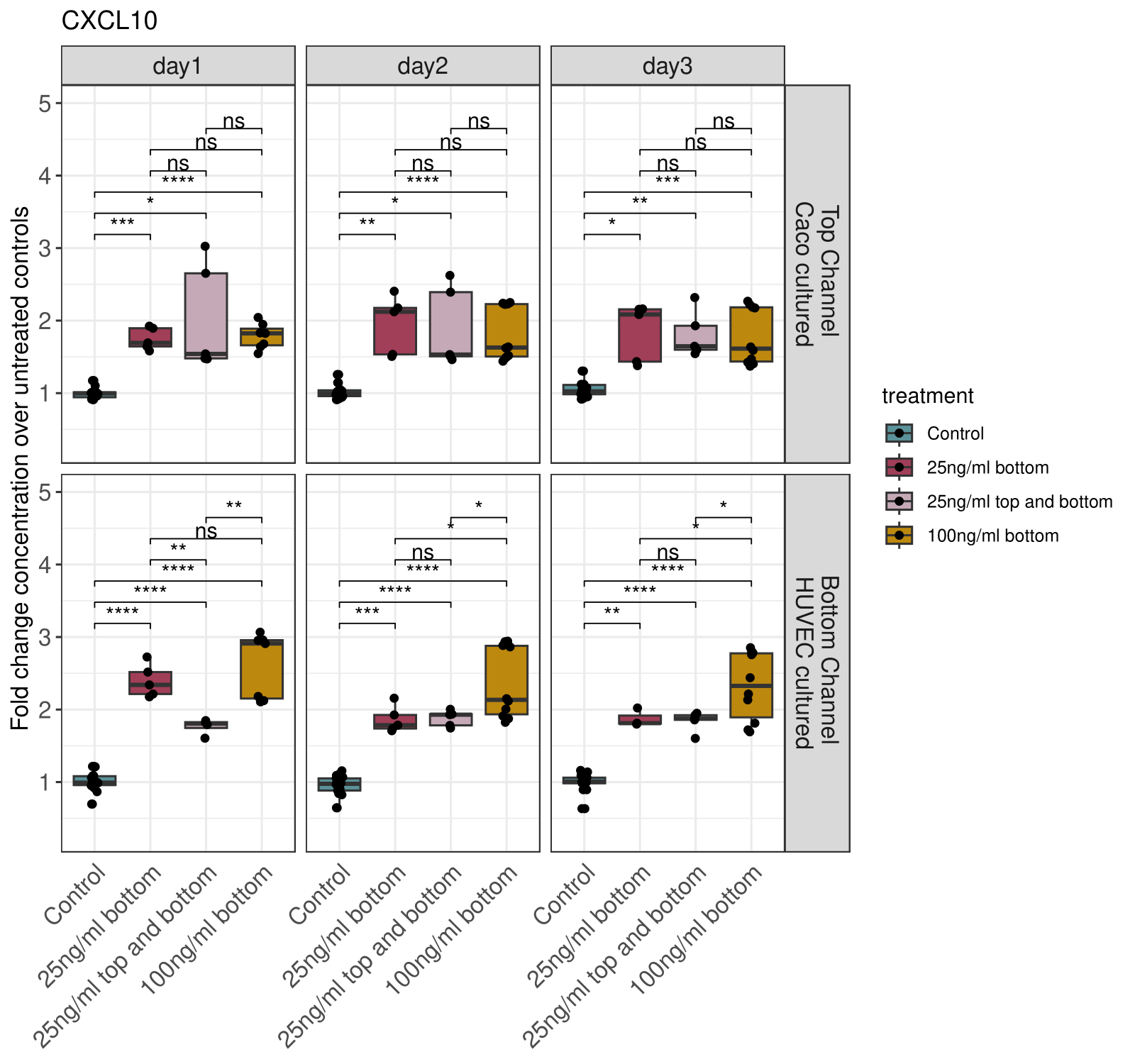

Supplement: S1 Fig — Increasing concentration of stimulating cytokine in bottom channel increased response in HUVECs but not Caco2s (red vs. yellow). Adding stimulating cytokine in top channel had no measurable effect on either cell type (red vs. purple). P-values determined using Wilcox rank sum test (p < .05 = *, p < 0.01 = **, p < 0.001 = ***, p < 0.0001 = ****). (TIF) [file pone.0289314.s001.tif]

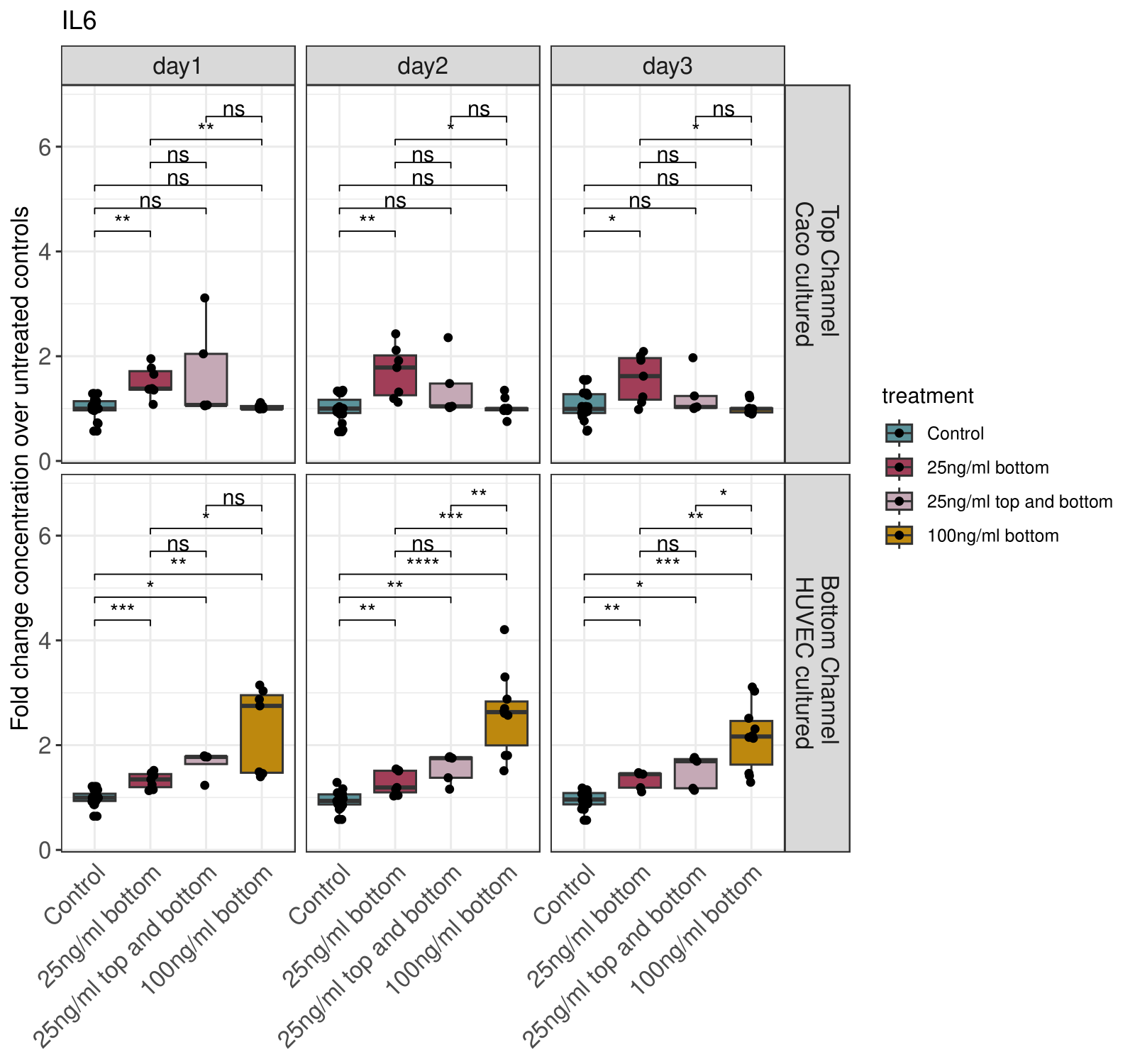

Supplement: S2 Fig — Increasing concentration of stimulating cytokine in bottom channel increased response in HUVECs but not Caco2s (red vs. yellow). Adding stimulating cytokine in top channel had no measurable effect on either cell type (red vs. purple). P-values determined using Wilcox rank sum test (p < .05 = *, p < 0.01 = **, p < 0.001 = ***, p < 0.0001 = ****). (TIF) [file pone.0289314.s002.tif]

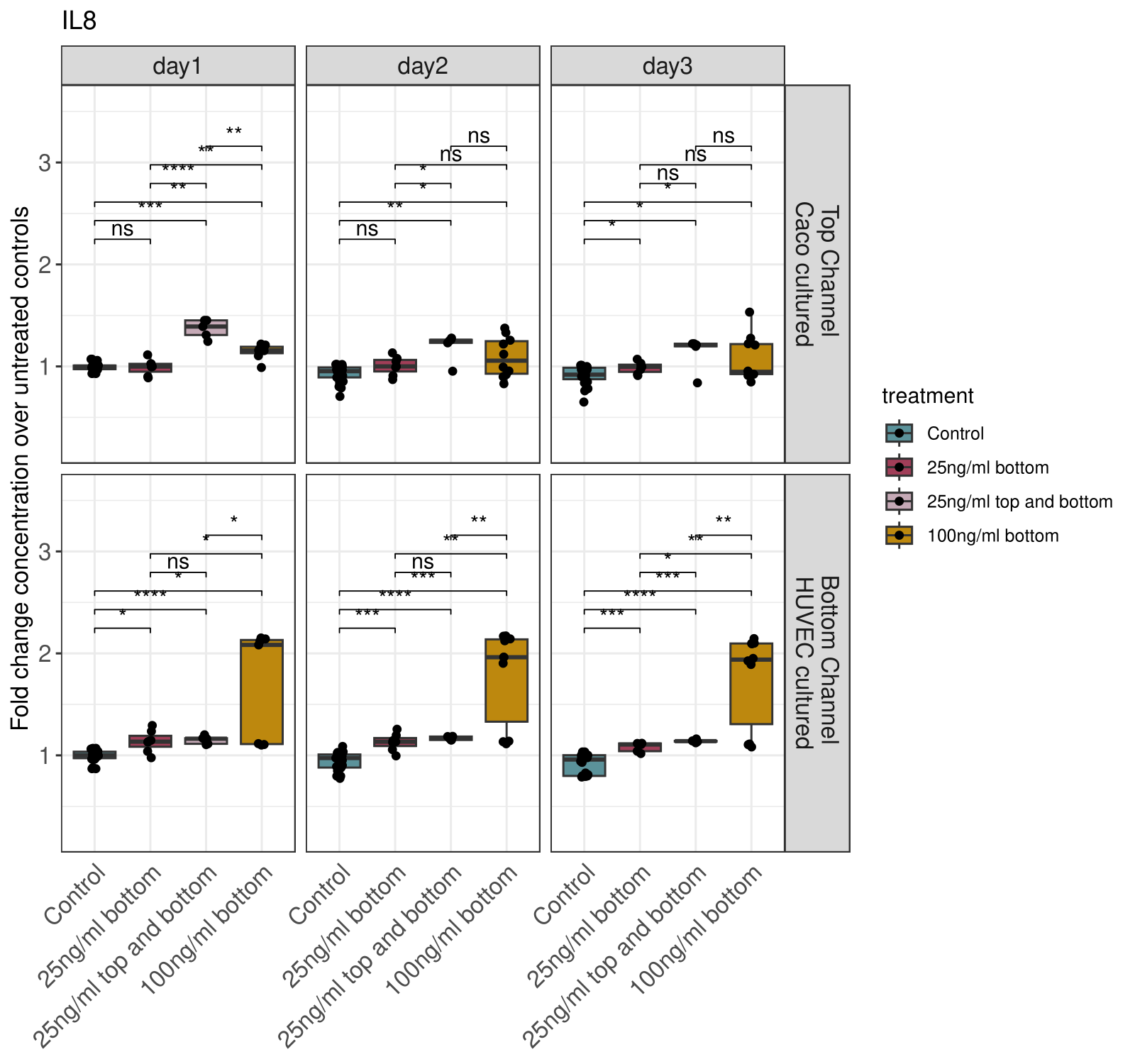

Supplement: S3 Fig — Increasing concentration of stimulating cytokine in bottom channel increased response in HUVECs but not Caco2s (red vs. yellow). Adding stimulating cytokine in top channel increased response in Caco2 cells but not HUVECs (red vs. purple). P-values determined using Wilcox rank sum test (p < .05 = *, p < 0.01 = **, p < 0.001 = ***, p < 0.0001 = ****. (TIF) [file pone.0289314.s003.tif]

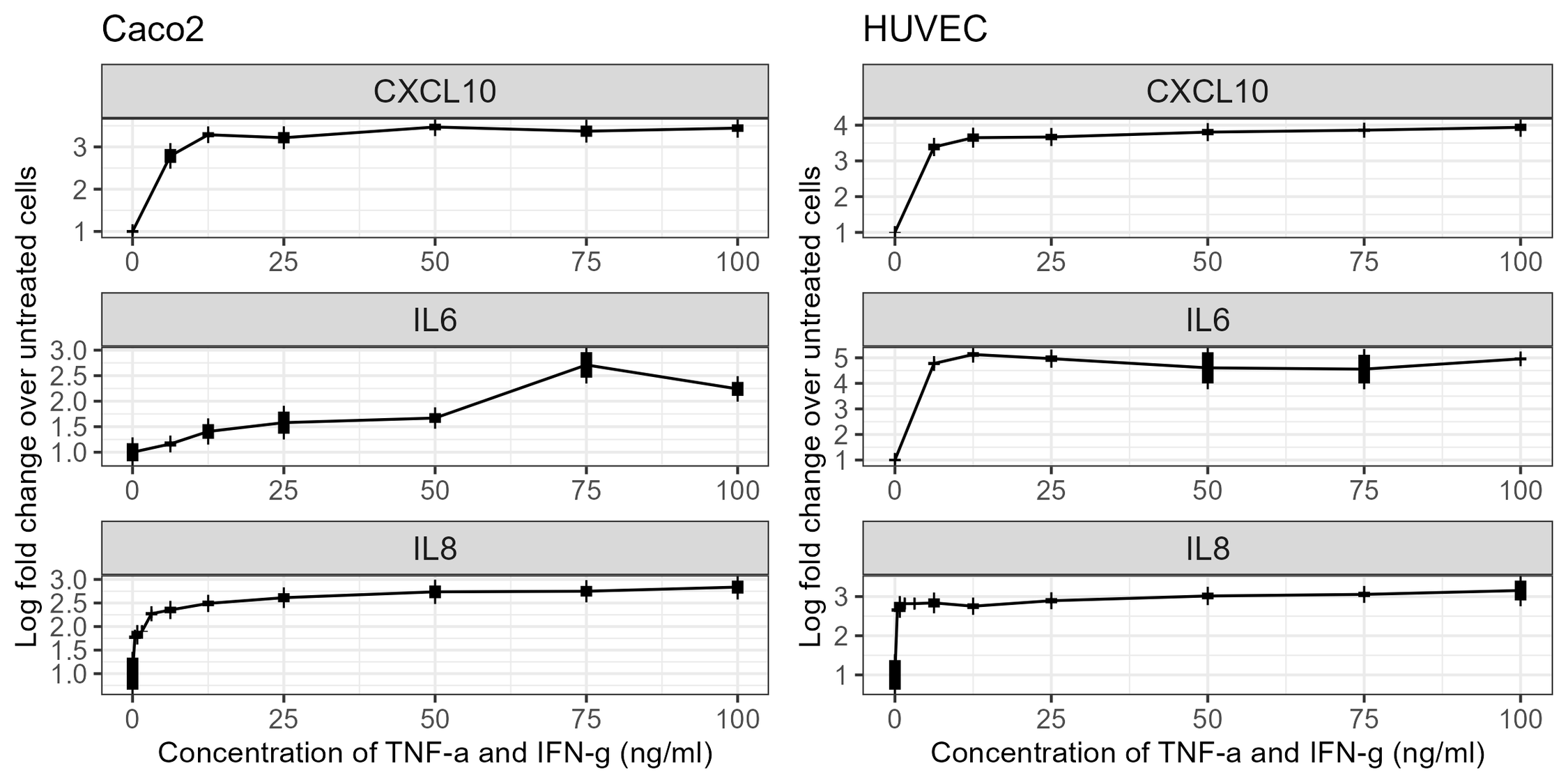

Supplement: S4 Fig — Maximum response is seen at concentrations of stimulating cytokine 25 ngml and above, with the exception of IL-6 in Caco2 cells. (TIF) [file pone.0289314.s004.tif]

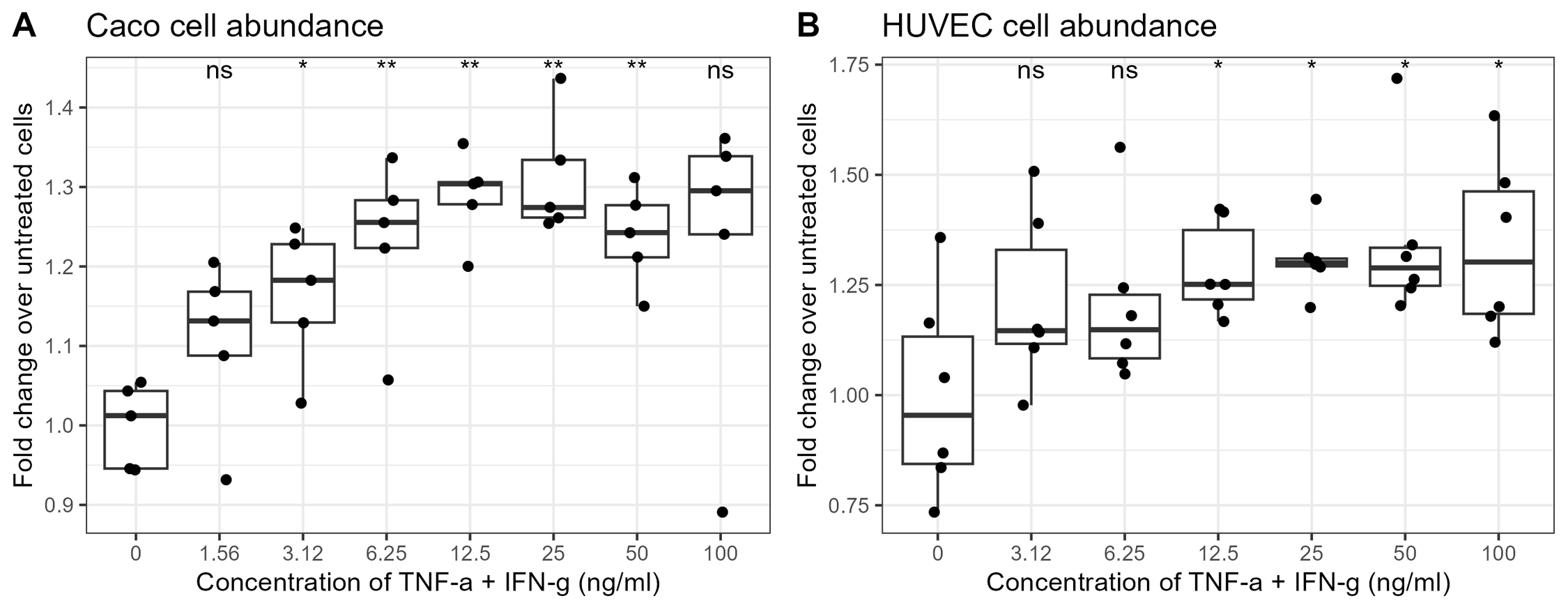

Supplement: S5 Fig — Neither cell type exhibits cell death compared to untreated cells. At some concentrations, cytokine treatment in fact increased cell growth and survival. P-values determined using Wilcox rank sum test over controls (p < .05 = *, p < 0.01 = **, p < 0.001 = ***, p < 0.0001 = ****). (TIF) [file pone.0289314.s005.tif]
